# *N*-[3,5-Dichloro-4-(1,1,2,2-tetra­fluoro­eth­oxy)phen­yl]-2,6-difluoro­benzamide

**DOI:** 10.1107/S1600536810029612

**Published:** 2010-07-31

**Authors:** Ying Liang, San Wei, Zi-Wen Yang

**Affiliations:** a Hubei Biopesticide Engineering Research Center, Hubei Academy of Agricultural Science, Wuhan 430064, People’s Republic of China

## Abstract

In the title compound, C_15_H_7_Cl_2_F_6_NO_2_, the conformation of the N—H bond in the amide segment is *anti* to the C=O bond and the dihedral angle between the two benzene rings is 78.6 (3)°. The terminal –CHF_2_ group is disordered over two orientations in a 0.67:0.33 ratio. In the crystal, the mol­ecules are linked by N—H⋯O hydrogen bonds, generating *C*(4) chains propagating in [100].

## Related literature

For background to the biological properties of related compounds, see: Liu, Li & Li (2004 ▶); Liu, Li & Zhong (2004 ▶); Shiga *et al.* (2003 ▶). For a related structure, see: Gowda *et al.* (2010 ▶). For reference structural data, see: Allen *et al.* (1987 ▶).
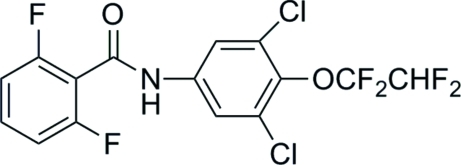


## Experimental

### 

#### Crystal data

C_15_H_7_Cl_2_F_6_NO_2_
*M* *_r_* = 418.12
Orthorhombic, 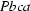

*a* = 9.426 (2) Å
*b* = 15.568 (4) Å
*c* = 22.601 (6) Å
*V* = 3316.7 (15) Å^3^
*Z* = 8
Mo *K*α radiation
μ = 0.47 mm^−1^
*T* = 298 K
0.16 × 0.12 × 0.10 mm

#### Data collection

Bruker SMART APEX CCD diffractometer
Absorption correction: multi-scan (*SADABS*; Bruker, 2000 ▶) *T*_min_ = 0.929, *T*_max_ = 0.955
16415 measured reflections
2916 independent reflections
2111 reflections with *I* > 2σ(*I*)
*R*_int_ = 0.075

#### Refinement

*R*[*F* ^2^ > 2σ(*F* ^2^)] = 0.084
*wR*(*F* ^2^) = 0.253
*S* = 1.06
2916 reflections
254 parameters
35 restraints
H-atom parameters constrained
Δρ_max_ = 0.54 e Å^−3^
Δρ_min_ = −0.41 e Å^−3^

### 

Data collection: *SMART* (Bruker, 2000 ▶); cell refinement: *SAINT* (Bruker, 2000 ▶); data reduction: *SAINT*; program(s) used to solve structure: *SHELXS97* (Sheldrick, 2008 ▶); program(s) used to refine structure: *SHELXL97* (Sheldrick, 2008 ▶); molecular graphics: *SHELXTL* (Sheldrick, 2008 ▶); software used to prepare material for publication: *SHELXTL*.

## Supplementary Material

Crystal structure: contains datablocks global, I. DOI: 10.1107/S1600536810029612/hb5568sup1.cif

Structure factors: contains datablocks I. DOI: 10.1107/S1600536810029612/hb5568Isup2.hkl

Additional supplementary materials:  crystallographic information; 3D view; checkCIF report

## Figures and Tables

**Table 1:** Hydrogen-bond geometry (Å, °)

*D*—H⋯*A*	*D*—H	H⋯*A*	*D*⋯*A*	*D*—H⋯*A*
N1—H1⋯O1^i^	0.86	2.00	2.861 (5)	174

Symmetry code: (i) 
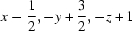
.

**Fig. 1.**
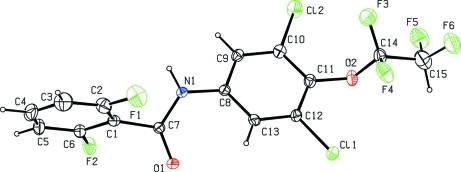
The structure of (I), showing 50% probability displacement ellipsoids.

**Fig. 2.**
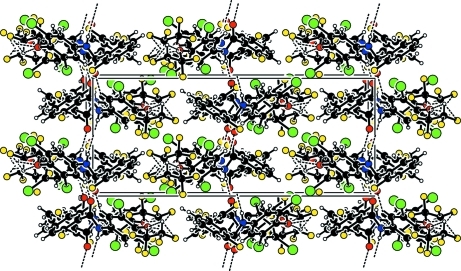
Crystal packing diagram of (I). Hydrogen bonds are shown as dashed lines.

## supplementary crystallographic information

### Comment

Amide derivatives showed diverse biological properties such as insecticidal (Liu, Li & Li, 2004), fungicidal (Liu, Li & Zhong, 2004) and acaricidal (Shiga *et al.*, 2003) activities. Commercialized compounds include benzamide (flutolanil, fluopicolide), nicotinamide (boscalid) and thiazole carboxamide (thifluzamide, ethaboxam). As a part of our study on the synthesis of new fluorine-containing compounds with possible biological activities, we report here the crystal structure of the title compound, (I)(Fig. 1).

In the molecule, all bond lengths and angles are normal (Allen *et al.*, 1987). The conformation of the N—H and the C=O bonds in the amide segment are anti to each other, which is similar to that observed in other amide compound (Gowda *et al.*, 2010). The dihedral angles between the two phenyl rings is 78.6°. The crystal structure is stabilized by intermolecular N—H···O hydrogen-bonds (Table 1).

### Experimental

Triethylamine (6 mmol) was added dropwise to a stirred solution of 3,5-dichloro-4-(1,1,2,2-tetrafluoroethoxy) aniline (5 mmol) and 2,6-dichlorobenzoyl chloride (5 mmol) in dry dichloromethane (20 ml) at 275–277 K. The mixture was stirred at 283–288 K for 2 h, then washed with 0.5% hydrochloric acid solution, and a saturated aqueous solution of sodium hydrogen carbonate, dried and evaporated. The residue was recrystallized from dichloromethane, giving colourless blocks of (I) after 3 weeks.

### Refinement

All H-atoms bound to carbon were refined using a riding model with d(C—H) = 0.93 Å, *U*_iso_=1.2*U*_eq_ (C) for aromatic 0.98 Å, *U*_iso_ = 1.2*U*_eq_ (C) for CH.

### Crystal data

**Table:**

C_15_H_7_Cl_2_F_6_NO_2_	*D*_x_ = 1.675 Mg m^−^^3^
*M**_r_* = 418.12	Mo *K*α radiation, λ = 0.71073 Å
Orthorhombic, *P**b**c**a*	Cell parameters from 3100 reflections
*a* = 9.426 (2) Å	θ = 2.7–20.5°
*b* = 15.568 (4) Å	µ = 0.47 mm^−^^1^
*c* = 22.601 (6) Å	*T* = 298 K
*V* = 3316.7 (15) Å^3^	Block, colorless
*Z* = 8	0.16 × 0.12 × 0.10 mm
*F*(000) = 1664	

### Data collection

**Table:**

Bruker SMART APEX CCD diffractometer	2916 independent reflections
Radiation source: fine-focus sealed tube	2111 reflections with *I* > 2σ(*I*)
graphite	*R*_int_ = 0.075
φ and ω scans	θ_max_ = 25.0°, θ_min_ = 2.6°
Absorption correction: multi-scan (*SADABS*; Bruker, 2000)	*h* = −11→11
*T*_min_ = 0.929, *T*_max_ = 0.955	*k* = −16→18
16415 measured reflections	*l* = −26→24

### Refinement

**Table:**

Refinement on *F*^2^	Primary atom site location: structure-invariant direct methods
Least-squares matrix: full	Secondary atom site location: difference Fourier map
*R*[*F*^2^ > 2σ(*F*^2^)] = 0.084	Hydrogen site location: inferred from neighbouring sites
*wR*(*F*^2^) = 0.253	H-atom parameters constrained
*S* = 1.06	*w* = 1/[σ^2^(*F*_o_^2^) + (0.1378*P*)^2^ + 2.4511*P*] where *P* = (*F*_o_^2^ + 2*F*_c_^2^)/3
2916 reflections	(Δ/σ)_max_ = 0.001
254 parameters	Δρ_max_ = 0.54 e Å^−^^3^
35 restraints	Δρ_min_ = −0.40 e Å^−^^3^

### Special details

**Table:**

Geometry. All e.s.d.'s (except the e.s.d. in the dihedral angle between two l.s. planes) are estimated using the full covariance matrix. The cell e.s.d.'s are taken into account individually in the estimation of e.s.d.'s in distances, angles and torsion angles; correlations between e.s.d.'s in cell parameters are only used when they are defined by crystal symmetry. An approximate (isotropic) treatment of cell e.s.d.'s is used for estimating e.s.d.'s involving l.s. planes.
Refinement. Refinement of *F*^2^ against ALL reflections. The weighted *R*-factor *wR* and goodness of fit *S* are based on *F*^2^, conventional *R*-factors *R* are based on *F*, with *F* set to zero for negative *F*^2^. The threshold expression of *F*^2^ > σ(*F*^2^) is used only for calculating *R*-factors(gt) *etc*. and is not relevant to the choice of reflections for refinement. *R*-factors based on *F*^2^ are statistically about twice as large as those based on *F*, and *R*- factors based on ALL data will be even larger.

### Fractional atomic coordinates and isotropic or equivalent isotropic displacement parameters (Å^2^)

**Table:**

	*x*	*y*	*z*	*U*_iso_*/*U*_eq_	Occ. (<1)
C1	0.2885 (5)	0.6858 (3)	0.5451 (2)	0.0619 (12)	
C2	0.2068 (8)	0.6985 (5)	0.5949 (3)	0.096 (2)	
C3	0.1779 (10)	0.6367 (8)	0.6345 (4)	0.143 (4)	
H3	0.1250	0.6481	0.6684	0.172*	
C4	0.2302 (11)	0.5553 (7)	0.6230 (4)	0.132 (4)	
H4	0.2102	0.5110	0.6493	0.158*	
C5	0.3106 (8)	0.5382 (4)	0.5739 (4)	0.103 (2)	
H5	0.3445	0.4831	0.5665	0.124*	
C6	0.3391 (6)	0.6032 (4)	0.5365 (3)	0.0741 (15)	
C7	0.3282 (4)	0.7581 (3)	0.5045 (2)	0.0564 (11)	
C8	0.2294 (4)	0.8573 (3)	0.4316 (2)	0.0559 (11)	
C9	0.1389 (5)	0.8554 (3)	0.3827 (2)	0.0613 (12)	
H9	0.0775	0.8093	0.3773	0.074*	
C10	0.1405 (5)	0.9217 (3)	0.3424 (2)	0.0636 (12)	
C11	0.2300 (6)	0.9916 (3)	0.3497 (2)	0.0672 (13)	
C12	0.3205 (5)	0.9913 (3)	0.3980 (3)	0.0701 (14)	
C13	0.3201 (5)	0.9259 (3)	0.4389 (3)	0.0683 (14)	
H13	0.3807	0.9280	0.4714	0.082*	
C14	0.1507 (7)	1.1246 (4)	0.3126 (3)	0.0855 (17)	
C15	0.1869 (11)	1.1839 (5)	0.2601 (4)	0.146 (3)	
H15	0.2893	1.1945	0.2585	0.175*	0.67
H15'	0.2111	1.1413	0.2298	0.175*	0.33
Cl1	0.43376 (19)	1.07768 (10)	0.40998 (10)	0.1141 (8)	
Cl2	0.0311 (2)	0.91643 (11)	0.28089 (7)	0.1026 (7)	
F1	0.1596 (6)	0.7801 (4)	0.6046 (2)	0.1471 (19)	
F2	0.4199 (4)	0.5898 (2)	0.48811 (18)	0.1020 (12)	
F3	0.0163 (5)	1.1038 (3)	0.3168 (3)	0.1456 (19)	
F4	0.1720 (6)	1.1679 (3)	0.3624 (2)	0.1382 (18)	
N1	0.2209 (4)	0.7900 (2)	0.47228 (18)	0.0605 (10)	
H1	0.1390	0.7667	0.4771	0.073*	
O1	0.4498 (3)	0.7837 (2)	0.5020 (2)	0.0816 (11)	
O2	0.2375 (4)	1.0559 (2)	0.30668 (17)	0.0788 (11)	
F6	0.1153 (11)	1.2577 (5)	0.2708 (4)	0.163 (3)	0.67
F5	0.1452 (10)	1.1438 (6)	0.2108 (4)	0.161 (3)	0.67
F5'	0.3088 (15)	1.2237 (11)	0.2688 (8)	0.157 (6)	0.33
F6'	0.0536 (18)	1.2059 (19)	0.2406 (12)	0.219 (10)	0.33

### Atomic displacement parameters (Å^2^)

**Table:**

	*U*^11^	*U*^22^	*U*^33^	*U*^12^	*U*^13^	*U*^23^
C1	0.051 (3)	0.069 (3)	0.066 (3)	−0.002 (2)	−0.004 (2)	0.006 (2)
C2	0.095 (4)	0.118 (6)	0.073 (4)	0.010 (4)	0.008 (3)	0.010 (4)
C3	0.139 (7)	0.207 (11)	0.084 (5)	−0.018 (8)	0.013 (5)	0.049 (7)
C4	0.142 (8)	0.139 (7)	0.115 (7)	−0.049 (6)	−0.032 (6)	0.069 (6)
C5	0.122 (6)	0.076 (4)	0.112 (6)	−0.022 (4)	−0.030 (5)	0.038 (4)
C6	0.075 (3)	0.065 (3)	0.082 (4)	−0.007 (3)	−0.013 (3)	0.011 (3)
C7	0.048 (2)	0.048 (2)	0.074 (3)	0.006 (2)	0.002 (2)	−0.003 (2)
C8	0.043 (2)	0.048 (2)	0.076 (3)	0.0048 (18)	0.007 (2)	0.000 (2)
C9	0.063 (3)	0.051 (3)	0.069 (3)	−0.005 (2)	0.010 (2)	−0.008 (2)
C10	0.072 (3)	0.061 (3)	0.058 (3)	0.003 (2)	0.009 (2)	−0.012 (2)
C11	0.071 (3)	0.053 (3)	0.077 (3)	0.007 (2)	0.015 (3)	−0.001 (2)
C12	0.058 (3)	0.046 (3)	0.105 (4)	0.002 (2)	0.001 (3)	0.008 (2)
C13	0.059 (3)	0.046 (3)	0.100 (4)	0.006 (2)	−0.015 (3)	0.000 (2)
C14	0.105 (5)	0.058 (3)	0.093 (4)	0.006 (3)	−0.008 (3)	0.002 (3)
C15	0.155 (5)	0.127 (5)	0.154 (5)	0.027 (4)	−0.017 (4)	0.016 (4)
Cl1	0.0934 (12)	0.0600 (9)	0.189 (2)	−0.0223 (7)	−0.0398 (12)	0.0268 (10)
Cl2	0.1460 (16)	0.0985 (12)	0.0632 (9)	−0.0185 (10)	−0.0191 (9)	−0.0054 (7)
F1	0.174 (5)	0.159 (4)	0.109 (3)	0.050 (4)	0.041 (3)	−0.021 (3)
F2	0.123 (3)	0.067 (2)	0.116 (3)	0.0233 (19)	0.022 (2)	0.0026 (18)
F3	0.097 (3)	0.097 (3)	0.243 (6)	0.021 (2)	0.027 (3)	0.002 (3)
F4	0.186 (5)	0.104 (3)	0.125 (3)	0.062 (3)	−0.023 (3)	−0.016 (3)
N1	0.042 (2)	0.055 (2)	0.084 (3)	−0.0037 (16)	0.0022 (18)	0.0098 (19)
O1	0.0474 (19)	0.064 (2)	0.134 (3)	−0.0028 (16)	−0.014 (2)	0.020 (2)
O2	0.091 (3)	0.059 (2)	0.086 (3)	0.0114 (18)	0.022 (2)	0.0166 (18)
F6	0.179 (5)	0.136 (4)	0.174 (5)	0.032 (4)	−0.008 (4)	0.038 (4)
F5	0.189 (5)	0.165 (5)	0.129 (4)	0.017 (4)	−0.020 (4)	0.012 (4)
F5'	0.157 (7)	0.154 (7)	0.160 (7)	−0.004 (5)	0.007 (5)	0.006 (5)
F6'	0.217 (11)	0.221 (11)	0.218 (11)	0.004 (5)	−0.006 (5)	0.005 (5)

### Geometric parameters (Å, °)

**Table:**

C1—C2	1.377 (8)	C10—Cl2	1.733 (5)
C1—C6	1.385 (7)	C11—C12	1.385 (7)
C1—C7	1.500 (7)	C11—O2	1.398 (6)
C2—C3	1.344 (11)	C12—C13	1.375 (7)
C2—F1	1.363 (8)	C12—Cl1	1.738 (5)
C3—C4	1.384 (13)	C13—H13	0.9300
C3—H3	0.9300	C14—F3	1.311 (8)
C4—C5	1.370 (13)	C14—F4	1.328 (7)
C4—H4	0.9300	C14—O2	1.352 (7)
C5—C6	1.346 (8)	C14—C15	1.540 (8)
C5—H5	0.9300	C15—F5'	1.321 (10)
C6—F2	1.350 (7)	C15—F5	1.335 (8)
C7—O1	1.215 (5)	C15—F6	1.355 (8)
C7—N1	1.341 (6)	C15—F6'	1.375 (10)
C8—C13	1.379 (6)	C15—H15	0.9800
C8—N1	1.395 (6)	C15—H15'	0.9791
C8—C9	1.398 (7)	N1—H1	0.8600
C9—C10	1.375 (7)	F5—H15'	0.7554
C9—H9	0.9300	F5'—H15	0.5415
C10—C11	1.388 (7)		
			
C2—C1—C6	116.1 (5)	C12—C13—C8	119.7 (5)
C2—C1—C7	122.1 (5)	C12—C13—H13	120.2
C6—C1—C7	121.6 (5)	C8—C13—H13	120.2
C3—C2—F1	119.6 (8)	F3—C14—F4	102.1 (6)
C3—C2—C1	123.7 (8)	F3—C14—O2	113.3 (5)
F1—C2—C1	116.7 (6)	F4—C14—O2	113.2 (5)
C2—C3—C4	117.3 (9)	F3—C14—C15	114.7 (6)
C2—C3—H3	121.4	F4—C14—C15	108.4 (6)
C4—C3—H3	121.4	O2—C14—C15	105.3 (6)
C5—C4—C3	121.8 (7)	F5'—C15—F5	126.8 (12)
C5—C4—H4	119.1	F5'—C15—F6	90.5 (10)
C3—C4—H4	119.1	F5—C15—F6	113.5 (9)
C6—C5—C4	118.2 (8)	F5'—C15—F6'	136.5 (16)
C6—C5—H5	120.9	F5—C15—F6'	65.2 (13)
C4—C5—H5	120.9	F6—C15—F6'	52.4 (12)
C5—C6—F2	120.3 (6)	F5'—C15—C14	111.1 (10)
C5—C6—C1	122.8 (7)	F5—C15—C14	107.3 (8)
F2—C6—C1	116.8 (4)	F6—C15—C14	105.1 (7)
O1—C7—N1	124.4 (4)	F6'—C15—C14	101.2 (13)
O1—C7—C1	120.6 (4)	F5'—C15—H15	21.3
N1—C7—C1	115.0 (4)	F5—C15—H15	109.8
C13—C8—N1	122.6 (5)	F6—C15—H15	110.7
C13—C8—C9	119.3 (4)	F6'—C15—H15	147.7
N1—C8—C9	118.1 (4)	C14—C15—H15	110.3
C10—C9—C8	120.1 (4)	F5'—C15—H15'	102.6
C10—C9—H9	120.0	F5—C15—H15'	33.9
C8—C9—H9	120.0	F6—C15—H15'	144.6
C9—C10—C11	121.1 (5)	F6'—C15—H15'	99.0
C9—C10—Cl2	119.3 (4)	C14—C15—H15'	100.6
C11—C10—Cl2	119.6 (4)	H15—C15—H15'	82.0
C12—C11—C10	117.7 (5)	C7—N1—C8	126.3 (4)
C12—C11—O2	121.3 (5)	C7—N1—H1	116.9
C10—C11—O2	120.6 (5)	C8—N1—H1	116.9
C13—C12—C11	122.1 (5)	C14—O2—C11	117.9 (4)
C13—C12—Cl1	118.0 (4)	C15—F5—H15'	46.3
C11—C12—Cl1	119.9 (4)	C15—F5'—H15	41.2
			
C6—C1—C2—C3	1.5 (10)	C10—C11—C12—Cl1	−179.3 (4)
C7—C1—C2—C3	−174.9 (7)	O2—C11—C12—Cl1	6.7 (7)
C6—C1—C2—F1	178.3 (5)	C11—C12—C13—C8	1.4 (8)
C7—C1—C2—F1	1.9 (9)	Cl1—C12—C13—C8	178.6 (4)
F1—C2—C3—C4	−179.0 (8)	N1—C8—C13—C12	−178.3 (5)
C1—C2—C3—C4	−2.3 (13)	C9—C8—C13—C12	−0.1 (7)
C2—C3—C4—C5	1.3 (14)	F3—C14—C15—F5'	159.9 (10)
C3—C4—C5—C6	0.4 (13)	F4—C14—C15—F5'	46.6 (12)
C4—C5—C6—F2	178.8 (6)	O2—C14—C15—F5'	−74.8 (11)
C4—C5—C6—C1	−1.2 (10)	F3—C14—C15—F5	−57.7 (10)
C2—C1—C6—C5	0.4 (8)	F4—C14—C15—F5	−171.1 (7)
C7—C1—C6—C5	176.8 (5)	O2—C14—C15—F5	67.5 (9)
C2—C1—C6—F2	−179.7 (5)	F3—C14—C15—F6	63.3 (10)
C7—C1—C6—F2	−3.3 (7)	F4—C14—C15—F6	−50.0 (10)
C2—C1—C7—O1	109.9 (6)	O2—C14—C15—F6	−171.4 (8)
C6—C1—C7—O1	−66.3 (7)	F3—C14—C15—F6'	9.5 (16)
C2—C1—C7—N1	−70.5 (7)	F4—C14—C15—F6'	−103.8 (15)
C6—C1—C7—N1	113.3 (5)	O2—C14—C15—F6'	134.8 (15)
C13—C8—C9—C10	−0.2 (7)	O1—C7—N1—C8	−0.8 (8)
N1—C8—C9—C10	178.0 (4)	C1—C7—N1—C8	179.6 (4)
C8—C9—C10—C11	−0.6 (7)	C13—C8—N1—C7	−33.8 (7)
C8—C9—C10—Cl2	178.1 (4)	C9—C8—N1—C7	148.1 (5)
C9—C10—C11—C12	1.8 (7)	F3—C14—O2—C11	−55.4 (7)
Cl2—C10—C11—C12	−177.0 (4)	F4—C14—O2—C11	60.3 (7)
C9—C10—C11—O2	175.9 (4)	C15—C14—O2—C11	178.5 (6)
Cl2—C10—C11—O2	−2.9 (6)	C12—C11—O2—C14	−94.2 (6)
C10—C11—C12—C13	−2.2 (8)	C10—C11—O2—C14	92.0 (6)
O2—C11—C12—C13	−176.2 (4)		

### Hydrogen-bond geometry (Å, °)

**Table:**

*D*—H···*A*	*D*—H	H···*A*	*D*···*A*	*D*—H···*A*
N1—H1···O1^i^	0.86	2.00	2.861 (5)	174

Symmetry codes: (i) *x*−1/2, −*y*+3/2, −*z*+1.

## References

[bb1] Allen, F. H., Kennard, O., Watson, D. G., Brammer, L., Orpen, A. G. & Taylor, R. (1987). *J. Chem. Soc. Perkin Trans. 2*, pp. S1–19.

[bb2] Bruker (2000). *SMART*, *SAINT* and *SADABS* Bruker AXS Inc., Madison, Wisconsin, USA.

[bb3] Gowda, B. T., Tokarčík, M., Shakuntala, K., Kožíšek, J. & Fuess, H. (2010). *Acta Cryst.* E**66**, o1529–o1530. 10.1107/S1600536810019999 PMC3006934 21587779

[bb4] Liu, C. L., Li, L. & Li, Z. M. (2004). *Bioorg. Med. Chem.* **12**, 2825–2830. 10.1016/j.bmc.2004.03.050 15142542

[bb5] Liu, C. L., Li, Z. M. & Zhong, B. (2004). *J. Fluorine Chem.* **125**, 1287–1290.

[bb6] Sheldrick, G. M. (2008). *Acta Cryst.* A**64**, 112–122. 10.1107/S0108767307043930 18156677

[bb7] Shiga, Y., Okada, I. & Fukuchi, T. (2003). *J. Pestic. Sci.* **28**, 310–312.

